# Vascular density with optical coherence tomography angiography and systemic biomarkers in low and high cardiovascular risk patients

**DOI:** 10.1038/s41598-020-73861-z

**Published:** 2020-10-07

**Authors:** Marc-Antoine Hannappe, Louis Arnould, Alexandre Méloux, Basile Mouhat, Florence Bichat, Marianne Zeller, Yves Cottin, Christine Binquet, Catherine Vergely, Catherine Creuzot-Garcher, Charles Guenancia

**Affiliations:** 1 Ophthalmology Department, University Hospital, 14 rue Paul Gaffarel, 21079 Dijon Cedex, France; 2 Laboratoire de Physiopathologie et Epidémiologie Cérébro-Cardiovasculaires (EA7460, PEC2), UFR Des Sciences de Santé, Bourgogne Franche-Comté University, Dijon, France; 3 Centre des Sciences du Goût et de l’Alimentation, AgroSup Dijon, CNRS, INRAE, Université Bourgogne Franche-Comté, 21000 Dijon, France; 4 INSERM, CIC1432, Clinical Epidemiology Unit, Dijon, France; 5 Dijon University Hospital, Clinical Investigation Center, Clinical Epidemiology/Clinical Trials Unit, Dijon, France; 6 Cardiology Department, University Hospital, Dijon, France

**Keywords:** Anatomy, Biomarkers

## Abstract

We aimed to compare retinal vascular density in Optical Coherence Tomography Angiography (OCT-A) between patients hospitalized for acute coronary syndrome (ACS) and control patients and to investigate correlation with angiogenesis biomarkers. Patients hospitalized for an acute coronary syndrome (ACS) in the Intensive Care Unit were included in the “high cardiovascular risk” group while patients without cardiovascular risk presenting in the Ophthalmology department were included as “control”. Both groups had blood sampling and OCT-A imaging. Retina microvascularization density in the superficial capillary plexus was measured on 3 × 3 mm angiograms centered on the macula. Angiopoietin-2, TGF-β1, osteoprotegerin, GDF-15 and ST-2 were explored with ELISA or multiplex method. Overall, 62 eyes of ACS patients and 42 eyes of controls were included. ACS patients had significantly lower inner vessel length density than control patients (p = 0.004). A ROC curve found that an inner vessel length density threshold below 20.05 mm^−1^ was moderately associated with ACS. Significant correlation was found between serum levels of angiopoietin-2 and osteoprotegerin, and retinal microvascularization in OCT-A (R = − 0.293, p = 0.003; R = − 0.310, p = 0.001). Lower inner vessel length density measured with OCT-A was associated with ACS event and was also correlated with higher concentrations of angiopoietin-2 and osteoprotegerin.

## Introduction

In spite of the improvements in diagnosis and treatment of cardiovascular diseases (CVD), aging of population and urbanization make CVD one of the world’s major disease burdens^1,2^. Indeed, CVD remain a main cause of premature deaths and disability worldwide, with an estimated 16.7 million deaths in 2010, and projections show an overwhelming 23.3 million by 2030^3^.

Cardiovascular risk factors such as hypertension, hypercholesterolemia, and diabetes mellitus lead to systemic inflammation, vascular and cardiac oxidative stress, which contribute to coronary dysfunction and microvascular impairment^4^. Thus, coronary macro and microvascular alterations are closely associated and together contribute to the pathophysiology of myocardial ischemia^5^. The assessment of myocardial microvascularization is then of major interest in order to estimate the risk of acute coronary events; however, only invasive procedures, using intra vascular contrast agents, are currently available^6^.

Conversely, retinal microvascularization is easy to assess and can be studied through non-invasive exams^7^. Additionally, the retinal vascular network seems to reflect the systemic vascular status, and its study may even predict cardiovascular mortality^8–12^. Indeed, changes in the retinal vasculature, such as narrowing or tortuosity, have been associated with elevated blood pressure, heart failure or arterial stiffness index^9–13^.

Optical Coherence Tomography-Angiography (OCT-A) is a non-invasive method used to obtain detailed imaging of retina and choroidal vascularization without intravascular dye^14^. This device allows the quantitative evaluation of vascular parameters, such as the superficial capillary plexus (SCP) vessel density and foveal avascular zone (FAZ) area^15^. We previously showed in a pilot study that inner vessel length density in the SCP measured with OCT-A was associated with cardiovascular risk profiles in patients hospitalized for an acute coronary syndrome (ACS)^16^. However, these preliminary results need more supporting evidence with control patients. Moreover, histological analysis confirming the link between reduced vessel density in retina and myocardium is difficult and invasive^17^. Thus in this study, we aimed to reinforce our analysis of systemic microvascularization through biomarkers of angiogenesis and inflammation (angiopoietin-2, TGF-β1, osteoprotegerin, GDF-15 and ST-2). The purpose of this study was therefore to compare and try to seek for associations between retinal microvascularization with OCT-A and systemic microvascularization biomarkers in patients with a high cardiovascular risk profile and control subjects.

## Results

### Study population

**Figure 1:**
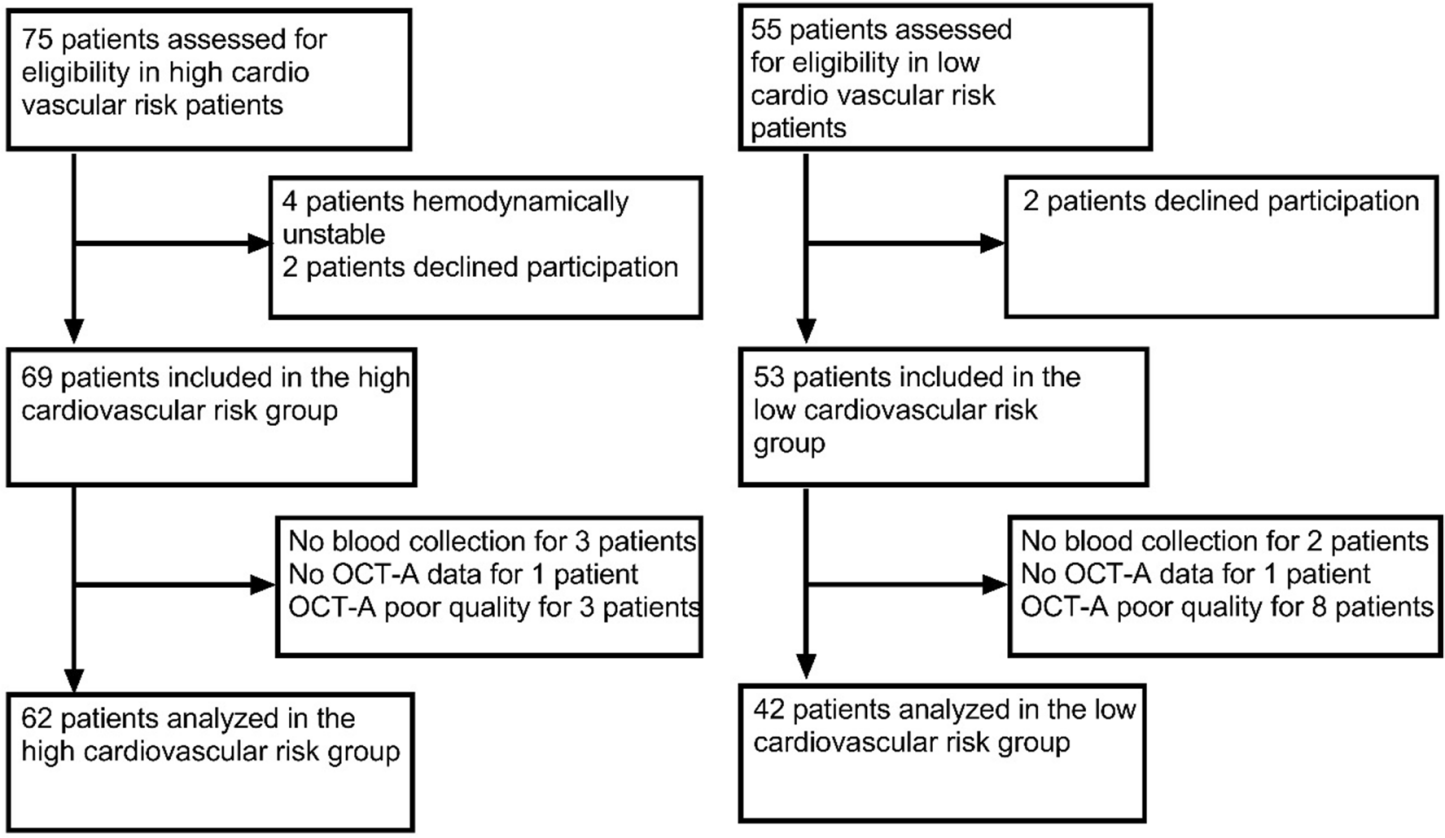
Flow chart of the high and low cardiovascular risk groups of patients.

We included 69 eyes of ACS patients from Cardiac Intensive Care Units and 53 eyes of patients from the Ophthalmology department. Eighteen eyes were excluded because their OCT-A exams were of poor quality or blood sample collection was not performed. Finally, 62 eyes of ACS patients and 42 eyes of control patients were kept for analysis (Fig. 1). The two groups were similar in age (p = 0.500) but there were less males in control group (p = 0.026). In the high cardiovascular risk group, 30 patients had hypertension (48%), 8 had diabetes (13%), 23 were smokers and had hypercholesterolemia (37%), 24 were obese (39%). Forty patients (65%) had a ST-segment Elevation Myocardial Infarction (STEMI) (Table 1).

**Table 1:** Patients baseline characteristics between acute coronary syndrome patients and controls.

	Acute coronary syndrome patients (n = 62)	Control patients (n = 42)	p value^a^
Age, years	61 (55–71)	61 (50–73)	0.5
Male	50 (81%)	25 (60%)	0.026
Hypertension	30 (48%)	0	
Diabetes	8 (13%)	0	
Hypercholesterolemia	23 (37%)	0	
Smoking	23 (37%)	0	
BMI > 30 kg/m^2^	24 (39%)	0	
STEMI	40 (65%)	0	
Axial length (mm)	23.75 ± 0.16	23.50 ± 0.27	0.490
Signal strength	8.7 ± 0.1	9.0 ± 0.1	0.080

*STEMI* ST-segment Elevation Myocardial Infarction. The continuous variables were expressed as Mean ± Standard Deviation, the dichotomous variables were expressed as numbers n (%).

^a^p values compared between the Acute Coronary Syndrome and control groups.

### Retinal vascular density analysis

Table 2 shows the quantitative data for retinal microvasculature assessed with OCT-A. There was a significant difference in retinal vascular density between ACS and control patients. Inner vessel length density in ACS patients was lower than in control subjects, respectively 19.76 mm^−1^ ± 1.77 and 20.67 mm^−1^ ± 1.33 (p = 0.004). A significant difference was also found for the inner perfusion density between ACS and control patients with respectively a mean measured at 0.360 ± 0.027 and 0.373 ± 0.024 (p = 0.011).

**Table 2:** Optical coherence tomography-angiography data characteristics in the superficial capillary plexus between acute coronary syndrome patients and control patients.

	Acute coronary syndrome patients (n = 62)	Control patients (n = 42)	p value^a^
FAZ (mm^2^)	0.21 ± 0.11	0.21 ± 0.11	0.949
Inner vessel length density (mm^−1^)	19.76 ± 1.77	20.67 ± 1.33	0.004
Full vessel length density (mm^−1^)	18.65 ± 1.84	19.51 ± 1.46	0.012
Inner perfusion density ([white pixels/(white + black pixels)] × 100)	0.36 ± 0.03	0.37 ± 0.02	0.011
Full perfusion density ([white pixels/(white + black pixels)] × 100)	0.34 ± 0.03	0.35 ± 0.03	0.016

The variables were expressed as Mean ± Standard Deviation.

*FAZ* Foveal Avascular Zone.

^a^p values compared between the Acute Coronary Syndrome and control groups.

**Figure 2:**
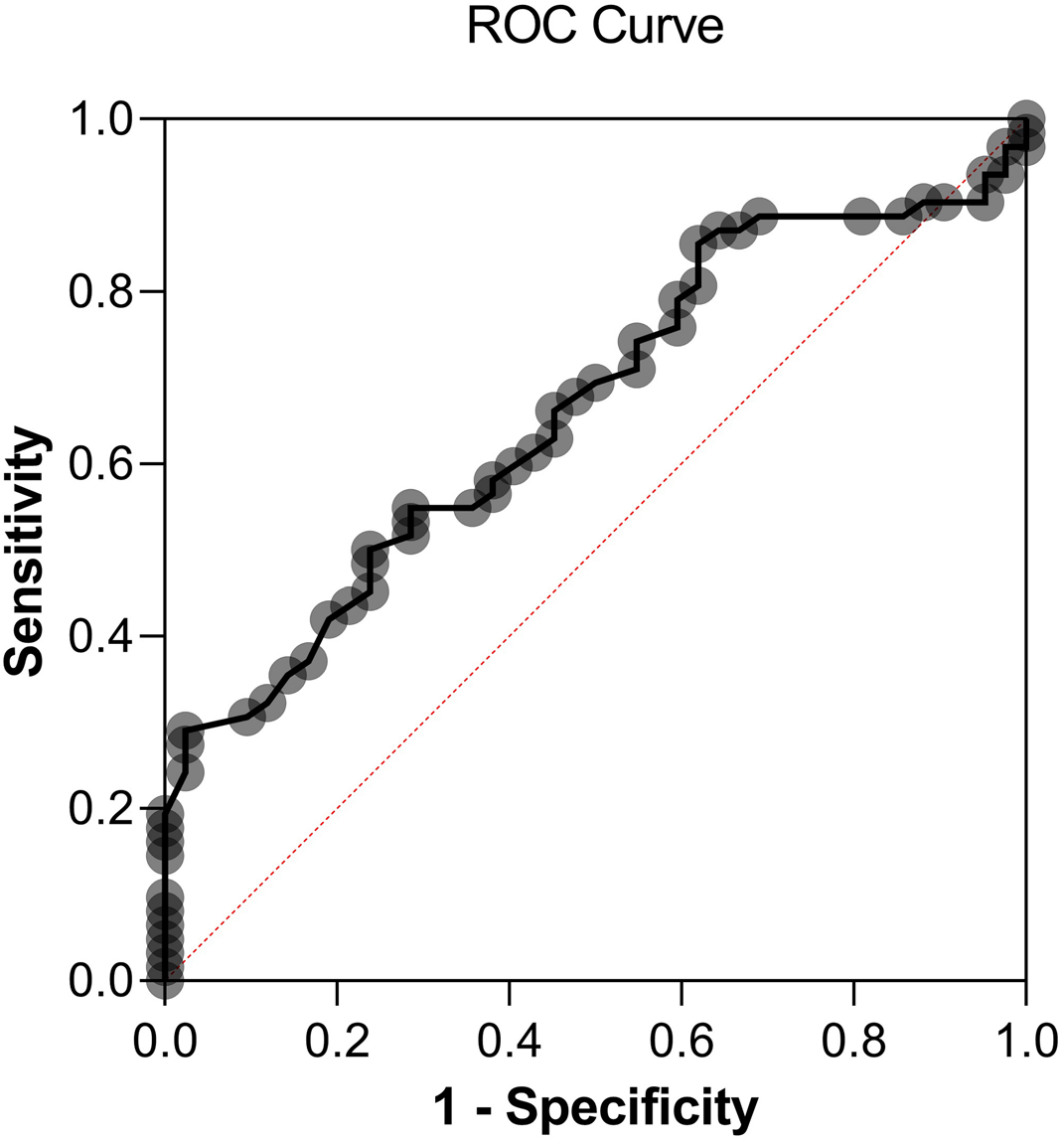
ROC curve showing sensitivity and specificity of inner vessel length density measured with Optical Coherence Tomography-Angiography for the occurrence of Acute Coronary Syndrome. Area under the ROC curve = 0.662; 95% CI 0.559–0.766; p < 0.001. An inner vessel length density value lower than 20.05 mm-1 was associated with ACS, with a specificity of 64% and a sensibility of 55%.

The analysis of ROC curve demonstrated the performance of the inner vessel length density to classify patients in the high cardiovascular group (area under the ROC curve 0.662; 95% CI 0.559–0.766; p < 0.001). From this ROC curve analysis (Fig. 2), an inner vessel length density value lower than 20.05 mm^−1^ was associated with ACS, with a specificity of 64% and a sensibility of 55%.

### Angiogenesis biomarkers analysis and correlations

**Figure 3:**
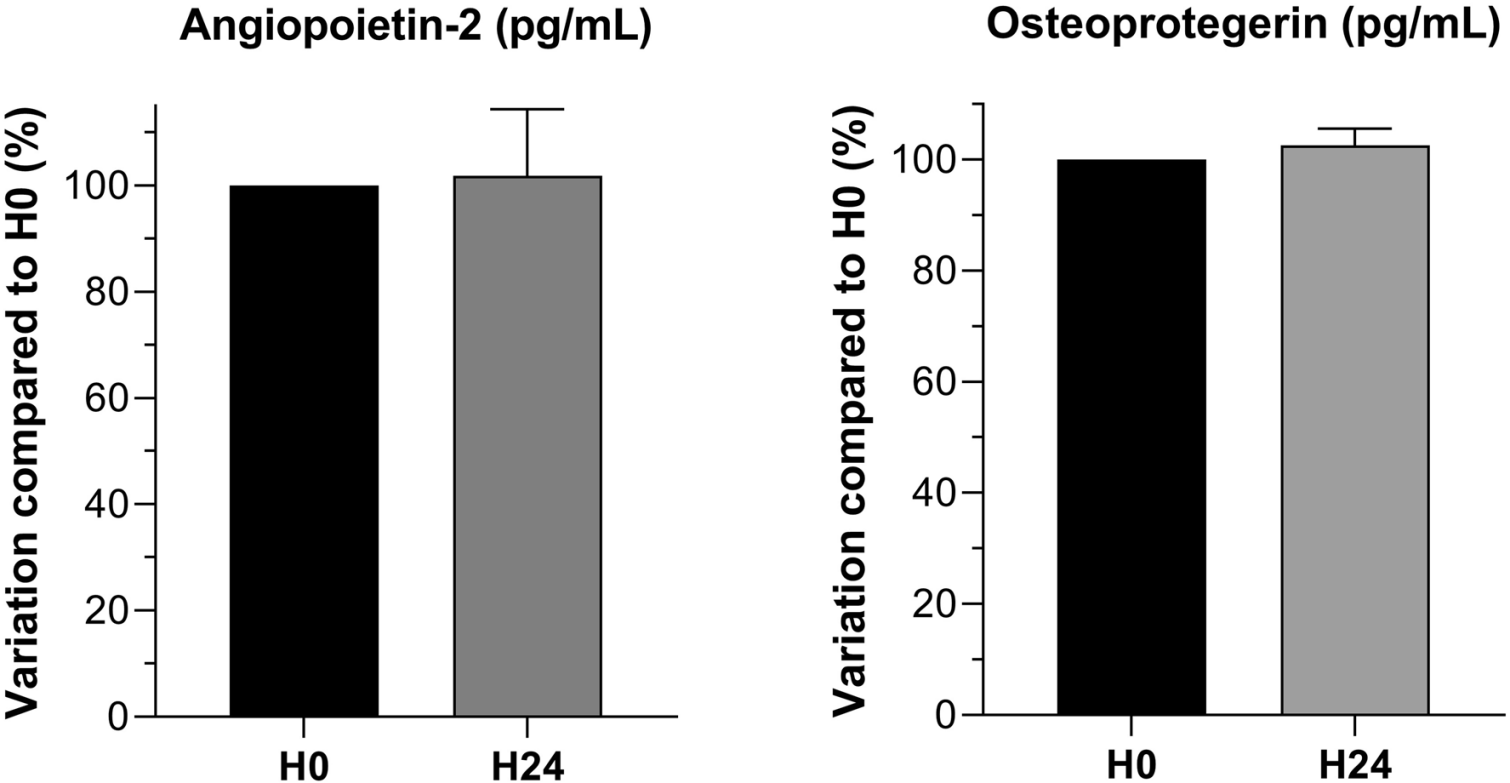
Osteoprotegerin and angiopoietin-2 stability tests at H24: percentage of the value of the H0 sampling (%).

Our preliminary stability assays showed a good stability at 24 h compared to baseline either with ELISA method for TGF-β1 and angiopoietin-2 measurements and with multiplex method for GDF-15, osteoprotegerin and ST-2 (Fig. 3).

**Figure 4:**
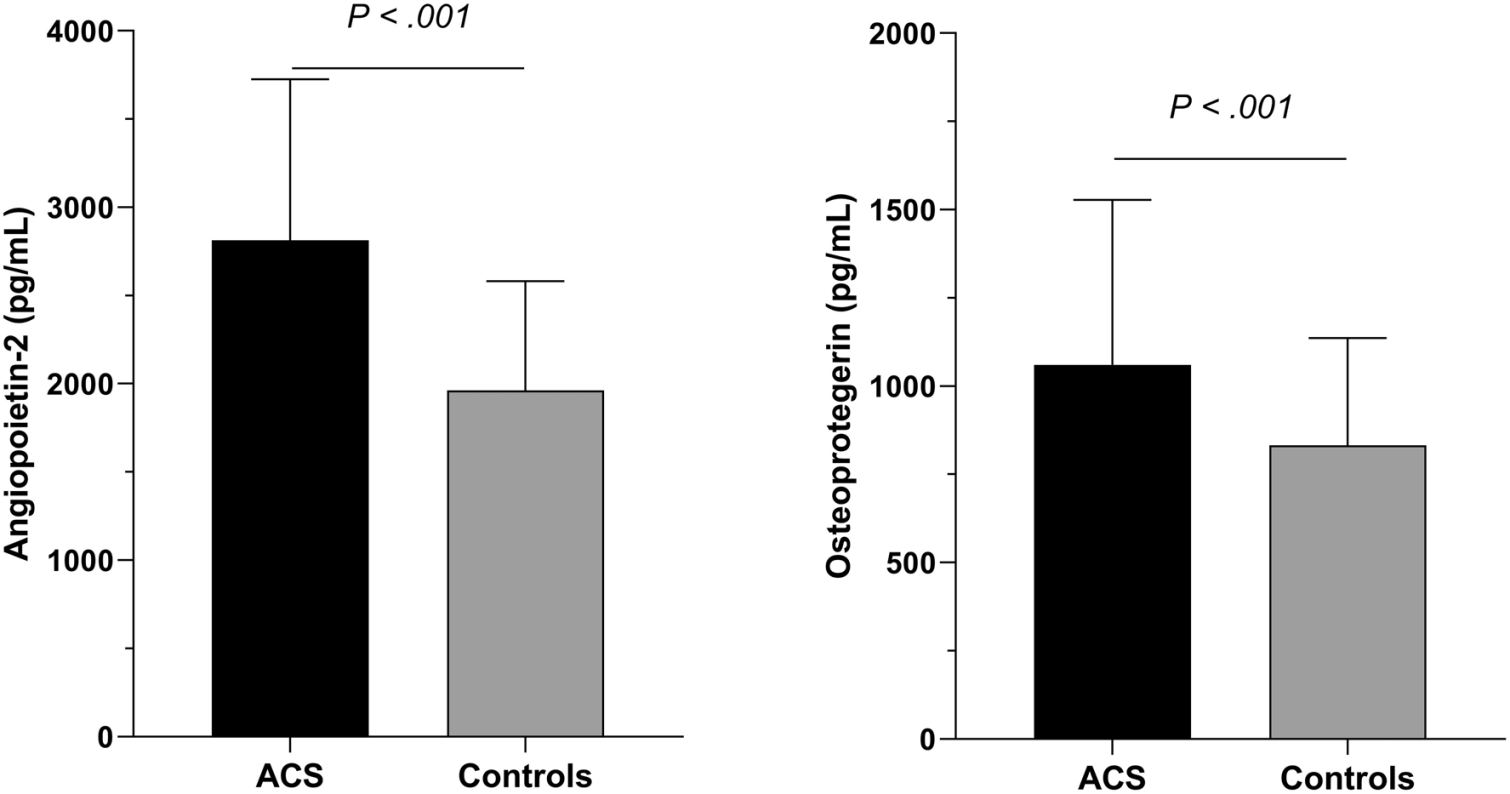
Comparison of angiopoietin-2 and osteoprotegerin blood levels between Acute Coronay Syndrome (ACS) patients and controls. Mean value ± Standard Deviation.

Serum levels of angiopoietin-2 and osteoprotegerin were significantly higher in ACS patients than controls with a mean respectively at 2814 ± 912 pg/mL versus 1962 ± 618 pg/mL (p < 0.001), and 1060 ± 467 pg/mL versus 828 ± 308 pg/mL (p = 0.006) (Fig. 4).

Serum levels of ST2 tended to be higher in ACS patients than controls with a mean respectively at 25,359 ± 42,250 pg/mL versus 13,435 ± 7,632 pg/mL (p = 0.074). No difference in TGF- β1 nor GDF-15 serum levels was found between ACS and control patients (respectively p = 0.700 and p = 0.240).

**Figure 5:**
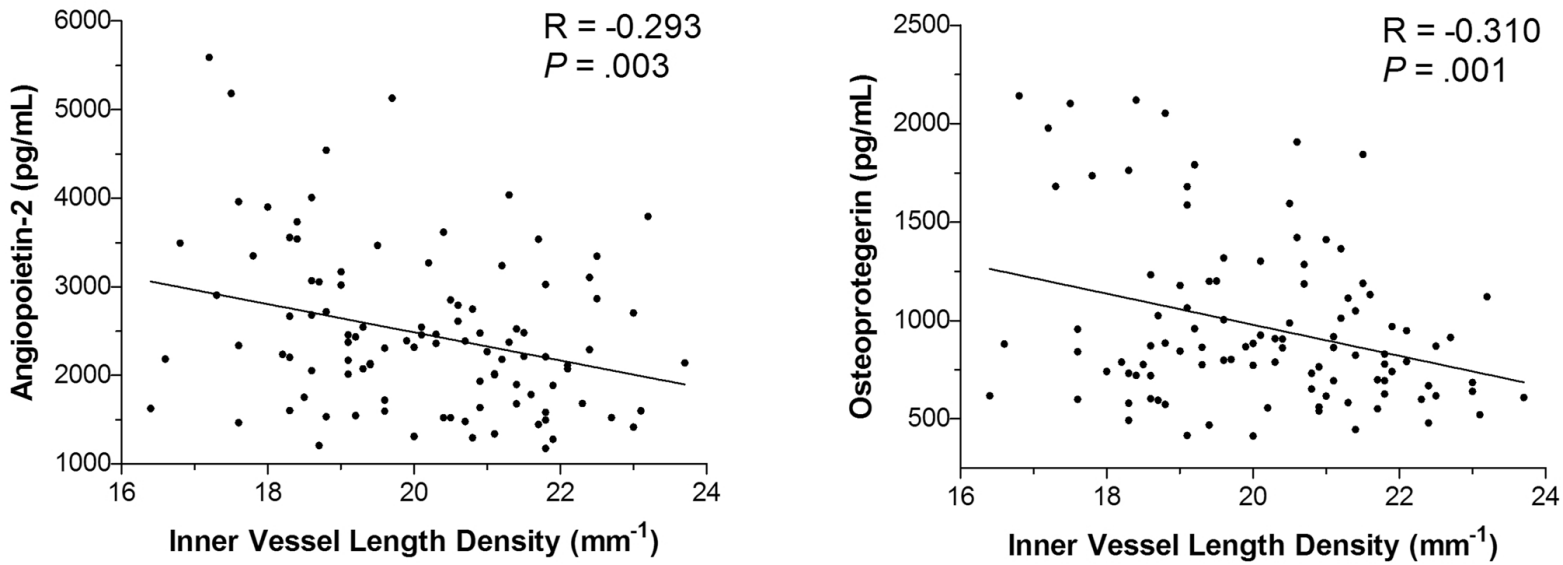
Correlation between blood levels of osteoprotegerin and angiopoietin-2 and inner vessel length density measured with OCT-A. A moderate correlation found that a higher rate of angiopoietin-2 or osteopretogerin was associated with a lower inner vessel length density.

Age was significantly correlated with inner vessel length density and inner perfusion density (respectively R = − 0.260, p = 0.008 and R = − 0.256, p = 0.009). A significant but moderate correlation was found between angiopoietin-2 and both inner vessel length density and inner perfusion density (respectively R = − 0.293, p = 0.003 and R = − 0.297, p = 0.002) (Fig. 5). Furthermore, osteoprotegerin showed a similar moderate correlation with inner vessel length density and inner perfusion density (respectively R = − 0.310, p = 0.001 and R = − 0.309, p = 0.001).

## Discussion

The main results of our study show that patients presenting with an ACS were more likely to have a lower inner vessel length density and that an inner vessel length density defined as less than 20.05 mm^−1^ was moderately associated with ACS. Moreover, angiopoetin-2 and osteoprotegerin circulating levels were associated with retinal vasculature parameters.

Our study showed that inner vessel length density assessed through OCT-A imaging was significantly lower in patients with ACS as compared to control patients matched for age. This result strengthens the hypothesis that retinal vasculature status does not act independently and can reflect a systemic cardio vascular alteration. Cheung et al. found that a narrower retinal arteriolar caliber was associated with left ventricular remodeling, independently of traditional risk factors and coronary atherosclerotic burden^18^. Seidelmann et al. also found that retinal vascular changes conferred long-term risk of mortality, ischemic stroke and coronary heart disease^9^. A low inner vessel length density evaluated with OCT-A was also involved in iodinated contrast agent induced acute kidney injury (AKI) following revascularization in ACS^19^. In obstructive sleep apnea–hypopnea syndrome patients, vessel densities in the parafoveal areas on OCT-A decreased with greater disease severity^20^. In the EYE-MI study, our group previously highlighted the association between inner vessel length density in OCT-A imaging and cardiovascular risk profile^16^. We also demonstrated that inner vessel length density was independent from systemic hemodynamic variables^21^. In the present study, patients presenting an ACS showed similar inner vessel length density as those in the EYE-MI study, measured respectively at 19.76 mm^−1^ and 19.70 mm^−1^. Our results support a link between low inner vessel length density measured with OCT-A and ACS. Moreover, we showed that an inner vessel length density below 20.05 mm^−1^ was moderately associated with ACS. A recent study also detected that inner vessel length density was decreased in patients with coronary heart disease compared to controls. Their findings were interesting because they correlated OCT-A data with coronary artery angiography and observed that greater coronary artery stenosis was negatively associated with retinal and choroidal vessel density^22^. This result is similar to what was found for diabetes mellitus in which OCT-A was used to identify pre-clinical diabetic retinopathy features^23^. Concerning FAZ analysis in our study, no difference was found in FAZ between ACS and control patients which can be explained by the fact this area varies considerably in healthy eyes^24^. Thus, it has been proposed that monitoring a single subject’s FAZ area enlargement in diabetic retinopathy is more informative in early stages of diabetic retinopathy than using measurements of FAZ alone^25^.

This study also showed a relation between OCT-A data and systemic biomarkers. The biomarkers chosen in this study were shown to be involved in angiogenesis and microvascular inflammation in both myocardium and retina^26–28^. Osteoprotegerin and its ligands system play an active role in pathological angiogenesis and inflammation in microvascularization^29^. They are involved in the endothelial homeostasis and muscular smooth cells alteration that leads to vascular calcification^29^. Thus, osteoprotegerin was associated with the risk of future coronary artery disease in apparently healthy men and women independently of established cardiovascular risk factors^30^. Angiopoietin-2 is a well-recognized vascular destabilizing factor, and higher levels of angiopoietin-2 were associated with decreased capillary density in a model of myocardial ischemia^31^. It appears to be a biomarker of poor outcome after ACS through exacerbating hypoxia and myocardial infarction^32^. In our study we showed that reduced inner vessel length density measured with OCT-A was associated with higher levels of osteoprotegerin and angiopoietin-2 blood levels. These correlations remained moderate regarding the sample size of our study. These findings highlight a link between retinal vascular density measurements made with OCT-A and blood circulating factors. Therefore, these results suggest a pathophysiological rationale to our hypothesis that retinal capillary rarefaction in OCT-A can be considered as a good reflect of systemic cardiovascular alteration. OCT-A analysis is not only beneficial for providing imaging of retina microvascularization but can also be associated with angiogenesis biomarkers modification. Thus, OCT-A could be a useful parameter to consider together with other pre-existing cardiovascular risk factors to assess high cardiovascular risk profiles and improve the screening of these patients in primary prevention.

We acknowledge several limitations to this study. First, many patients had to be excluded due to poor OCT-A quality^33^. Second, only participants able to have a retinal examination were included. Patients with the most severe ACS were therefore not analyzed, which could have introduced a selection bias. Third, the groups’ gender differences could also constitute a selection bias. Fourth, concerning biomarkers analysis, ACS is associated with a modification of systemic biomarkers kinetics which could be a confounding factor between retinal vascular density and biomarkers’ blood levels. Fifth, in our study, we collected ACS patients’ blood immediately after the admission in order to limit the effect of the acute cardiovascular event on the biomarkers’ kinetic. Indeed, significant modification of biomarkers’ kinetic after ACS have been shown to appear several hours or days after^32^. Moreover, kinetic biomarkers and their interpretation on vascularization is difficult to define due to their multiple interactions with each other and the clinical state of the tissue. Thus, the same biomarker can be pro-angiogenic or anti-angiogenic depending on the situation as demonstrated it with angiopoietin-2 in retina^34^. Sixth, in our study, we did not use axial length to scale individual images. In future studies, we should take into account axial length in order to correct for magnification error. Moreover, we should investigate the effect of signal strength and image processing method on our association^35^.

In conclusion, lower inner vessel length density measured with OCT-A was associated with ACS. Our findings also suggest a moderate correlation between angiopoietin-2 and osteoprotegerin and inner vessel length density. Thus, OCT-A seems to be a promising and useful assessment tool to assess cardiovascular risk, reflecting systemic microvascularization’s status. The clinical relevance of these findings deserves further studies.

## Methods

### Design and population of the study

This cross-sectional study included patients from the Coronary Care Unit and the Ophthalmology department of Dijon University Hospital from November 2017 to May 2019. Two groups of patients were assessed: high cardiovascular risk (ACS patients) and controls without self-declared cardiovascular risk (Ophthalmology patients). The study complied with the Declaration of Helsinki, Dijon University Hospital ethics committee gave prospective approval for the research protocol (N°2017-A02095-48, ClinicalTrials.gov identifier: NCT03551717) and written informed consent was obtained from every patient. This study followed the STROBE statement according to the EQUATOR (Enhancing the QUAlity and Transparency Of health Research) guidelines^36^.

Patients presenting with ACS, with or without ST-segment elevation, were included in the high cardiovascular risk group if their health condition allowed them to perform an ophthalmologic consultation. Patients older than 40 years old without cardiovascular risk (no diabetes, no arterial hypertension, no smoking, no previous cardiovascular event) presenting for a cataract surgery or refractive consultation were included from the Ophthalmology department. The non-inclusion criteria were: preexisting retinal disease (vascular and degenerative macular diseases, glaucoma, diabetic retinopathy, epiretinal membrane), patients under guardianship, patients without national health insurance, patients who refused to take part in the study, and patients with ongoing hemodynamic instability. We excluded myopic eyes with axial length > 26 mm because of retinal microvascular density modification with ocular elongation^37^. Patients from the Cardiology department benefited from an examination of the retinal microvasculature using OCT-A within the first forty eight hours of the cardiovascular event.

### Cardiovascular data and collection

In patients hospitalized for an ACS, data were collected from medical records of the RICO (obseRvatoire des Infarctus de Côte d’Or) survey. The design and methods of the RICO have been detailed previously^38^. In brief, age, sex, previous high blood pressure, previous diabetes, obesity, treated hypercholesterolemia, family history of coronary heart disease (CHD), smoking, personal cardiovascular and chronic kidney failure history, hemodynamic features and biological parameters (creatinine, blood glucose, HbA1c, troponin, Brain Natriuretic Peptid) were recorded.

### Description of retinal microvasculature with OCT-A

OCT-A was performed by technicians after mydriasis was obtained with tropicamide 0.5% (Thea, Clermond-Ferrand, France). During OCT-A exam, a cardiologist was present for monitoring patients’ heart rhythm and hemodynamic status if necessary. Axial length was measured using the IOLMaster 500 (Carl Zeiss Meditec, Lena, Germany). OCT-A images were obtained using a CIRRUS HD-OCT Model 5000 instrument (Carl Zeiss Meditec, Lena, Germany). The Angioplex v10 software was used to measure and collect retinal vascular features in the Superficial Capillary Plexus (SCP) and to measure the Foveal Avascular Zone (FAZ). We automatically measured two vascular densities with the Angioplex software: perfusion density and vessel length density. Perfusion density (area, percentage) represents the total area of perfused vasculature per unit area ([white pixels / (white + black pixels)] X 100), and vessel length density (length, mm − 1) represents the total length of perfused vasculature per unit area. All densities were measured in each sector of the SCP from the internal limiting membrane to the inner plexiform layer. The inner vessel length density and perfusion density are the average of the four sectors (superior, nasal, inferior sector and temporal sectors) in the SCP. We previously described these technical aspects^16^. The device automatically provides measurements for the foveal, parafoveal, and both areas together using the Early Treatment of Diabetic Retinopathy Study grid on en face OCT-A images. These regions are respectively presented as central, inner and full area in the Supplementary Fig. S1. In our study, we retained for analysis 3X3 mm angiograms centered on the macula. As quantitative studies on retinal microvasculature were shown to be repeatable^16,39–41^, the measurements were made on both eyes, but only one eye was retained for analysis according to the following procedure: (1) the angiogram with the highest quality of acquisition was retained; (2) images with a signal strength < 7/10 were excluded; (3) in single-eye patients, the functional eye was selected.

### Description of biomarkers pre-analytical protocol

Blood samples were collected at admission for Cardiology patients, and during OCT-A examination for Ophthalmology patients and allowed to clot into serum gel separation tubes. Serum was obtained after centrifugation and then separated into aliquots that were frozen at − 80 °C. Blood samples were stored at + 4 °C for a maximum of 24 h before centrifugation if necessary. Different biomarkers involved in angiogenesis and atherosclerosis were evaluated by enzyme-linked immunosorbent assay (ELISA Quantitine and Multiplex kits, Bio-Techne, Lille, France): angiopoietin-2, TGF-β1, osteoprotegerin, GDF-15 and ST-2. Indeed, angiogenic biomarkers such as angiopoietin-2 and TGF- β1 play a key role in physiology but also cardiovascular remodeling^42,43^. Osteoprotegerin, GDF-15 and ST-2 have been shown to represent independent biomarkers of cardiovascular diseases^44–46^.

Every assay was performed in duplicate, and a control inter-plate standard, composed of a mix of different patients’ serum, was used for standardization. Before taking any measurements in patients’ samples, we performed a stability assay to make sure the results were not affected by being stored for up to 24 h at 4 °C before centrifugation, sampling and freezing. For that purpose, 2 separate blood tubes were collected in ACS (n = 4) and controls patients (n = 4) and were centrifuged either within one hour after collection, or left 24 h at + 4 °C before processing. All biomarkers were analyzed for stability with appropriate methods in this study.

### Statistical analysis

Continuous variables were expressed as mean ± standard deviation (SD) or medians with interquartile range depending on their distribution. The dichotomous variables were expressed as numbers (percentages). For continuous variables, normality was verified with a Kolmogorov test. The unit was one eye per participant. In ACS patients’ clinical profiles, retina vasculature parameters measured through OCT-A, and serum biomarkers levels were assessed and compared with controls using a Student t-test for continuous variables and the chi-square or Fisher exact test for categorical variables. All tests were bilateral, and significance was set at p < 0.05. A Receiver Operating Characteristics (ROC) curve was used to find a predictable parameter associated with ACS. The correlation between the different biomarkers and OCT-A data was tested by a Pearson test. All analyses were performed using the SPSS 22.0 software (IBM, Chicago, IL, USA).

## Supplementary information

Supplementary Figure 1.
