# Supplementary material for: Vascular density with optical coherence tomography angiography and systemic biomarkers in low and high cardiovascular risk patients
Source: Sci Rep. 2020 Oct 7;10:16718. doi: 10.1038/s41598-020-73861-z (PMC7542456; doi:10.1038/s41598-020-73861-z)
Supplement: Supplementary file 1 — Supplementary Figure 1. [file 41598_2020_73861_MOESM1_ESM.docx]

**Vascular density with optical coherence tomography angiography and systemic biomarkers in low and high cardiovascular risk patients**

Marc-Antoine Hannappe^1,2^*, Louis Arnould^1,3,4^*, Alexandre Méloux^2,5^, Basile Mouhat^2,5^, Florence Bichat^2,5^, Marianne Zeller^2,5^, Yves Cottin^2,5^, Christine Binquet^4^, Catherine Vergely^2^, Catherine Creuzot-Garcher^1,4^, Charles Guenancia^2,5^


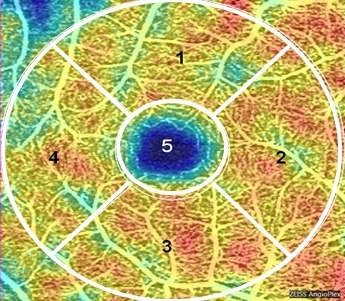


**Supplementary Figure S1**

Retinal vessel density of the Superficial Capillary Plexus on OCT-A (Carl Zeiss Meditec, Lena, Germany). The foveal and perifoveal area are divided into 5 sectors; 1: superior sector 2: nasal sector 3: inferior sector 4: temporal sector 5: central sector. Inner vessel density = 1+2+3+4; Full vessel density = 1+2+3+4+5.
